# Minimum-Entropy Optimal Control of Electromechanical Linkages for Energy Harvesting

**DOI:** 10.3390/e28050489

**Published:** 2026-04-24

**Authors:** Meysam Fathizadeh, Hanz Richter

**Affiliations:** Mechanical Engineering Department, Cleveland State University, Cleveland, OH 44115, USA; m.fathizadeh41@vikes.csuohio.edu

**Keywords:** energy harvesting, entropy-based cost functions, optimal control, mechanical–electrical power conversion

## Abstract

This work considers optimal mechanical–electrical power conversion across rigid linkages equipped with current-controlled actuators. A novel cost function derived from a generalization of the Second Law of Thermodynamics is adopted from our previous work, where cycle-averaged energies are interpreted as generalized temperatures. A cost function based on generalized entropy generation is used to formulate an optimal control problem yielding a decoupled velocity feedback controller. Suboptimal gains are found, which are independent of both the excitation characteristics and the mechanical subsystem dynamics, and yield closed-loop stability. The effectiveness and simplicity of the resulting controller is demonstrated by a Monte Carlo simulation study, where random episodes of unknown, periodic forcing are applied under the proposed controller and compared with a maximum-efficiency controller. Results show that the proposed controller offers a higher statistical expectation for the average harvested power.

## 1. Introduction

This paper introduces a novel systematic principle for the design and control of electromechanical power conversion systems. This includes energy harvesters featuring linkages with multiple degrees of freedom (DoFs), resembling rigid robots. Energy harvesting devices, for instance, wave energy converters (WECs), operate by transforming mechanical excitation from wave motion to electrical power, which is subsequently stored. A particular feature of this application is the fact that the external forces from which mechanical power is converted are not known in advance, and further, their spectral characteristics may be poorly understood and shifting with time.

Efficiency is a primary consideration for the design and operation of many engineering systems but may not be an adequate target in all applications. Efficiency is measured by the ratio of a product to the resource invested in obtaining it, which is often monetary. In applications such as energy harvesting from a free, inexhaustible, and renewable resource, efficiency may not be the primary consideration but rather the *absolute* amount of harvested power is of importance. As this paper shows, a suitable multidomain extension of the Second Law of Thermodynamics yields a cost function that may be conveniently used in the formulation of optimal control problems (OCPs) capturing the above considerations.

Energy harvesting has been extensively investigated, with a wide range of devices employing piezoelectric, triboelectric, and metamaterial technologies [1,2]. There are numerous concepts for harvesting wave energy, which vary widely in their operating principles. Among the many device types, the point-absorber wave energy converter (PAWEC) is considered one of the simplest, most versatile, and most promising approaches, and it has been the subject of extensive investigation worldwide [3].

Pendulum-based mechanisms are widely incorporated into ocean WEC devices. While single-pendulum designs are commonly found, they are designed to be effective over a narrow frequency range, matching an assumed spectrum of wave motion. A double pendulum, on the other hand, offers more opportunities for power harvesting due to the extended configuration space and the use of two generators. These devices have shown better performance under various wave frequencies [4,5]. Our work aims to study optimal electromechanical power conversion involving serial linkages of arbitrary configuration and degrees of freedom (DoFs) with electrical actuation. This objective is achieved by introducing a novel cost function based on the laws of thermodynamics.

The First Law of Thermodynamics (FLT), also referred to as the energy conservation principle, governs energy conversion across all physical domains and is the basis for defining efficiency. The Second Law of Thermodynamics (SLT) introduces further constraints on energy conversion in processes that satisfy the FLT. The methodologies of *Entropy Generation Minimization* (EGM) pioneered by Bejan [6] involve optimality criteria related to the SLT rather than common efficiency. The origin of the second law traces back to the work of Sadi Carnot, who proposed a thermodynamic cycle consisting of two isothermal and two adiabatic processes. He showed that this reversible cycle represents the maximum possible efficiency of any heat engine. Later, these ideas were formalized by introducing entropy and expressing the second law through Clausius’s inequality:

(1) $$\oint \frac{dQ}{T}\leq 0$$

where *Q* is the heat transfer and *T* is the temperature. Entropy became a central concept in thermodynamics, representing system disorder and efficiency loss. Gouy and Stodola later introduced the concepts of *entropy generation* and *lost work*, linking irreversibility and ambient temperature to thermodynamic efficiency [7]. This led to the *Gouy–Stodola theorem*:

(2) $$W_{lost}=T_{0}{\dot{S}}_{gen}$$

where $T_{0}$ is the ambient temperature and ${\dot{S}}_{gen}$ is the entropy generation rate due to irreversibilities. Extensive research has since focused on improving thermodynamic performance by minimizing lost work or entropy generation. For constant environmental temperature, these two objectives are equivalent [8,9].

Entropy production control can be pursued with different objectives. The EGM is typically desirable in systems involving work exchange, such as heat engines or refrigerators. However, EGM is not the only application of the entropy concept. The maximum entropy production principle (MEPP) has also been employed in specific classes of problems [10,11]. In [12], MEPP is incorporated into a reinforcement learning-based adaptive controller to enhance exploration of the learning space and improve the overall performance of an adaptive PID controller. Similarly, Martino [13] used MEPP to transform sparse and coarse biological data into probabilistic models by selecting the least biased distribution. These are examples of applications of the microscopic definition of entropy, whereas the present study adopts the macroscopic view of entropy as defined in classical thermodynamics.

Unlike the FLT, SLT principles and the limits it places on power conversion performance are not immediately or easily applicable outside classical thermodynamics. Readers are referred to the work of Haddad [14] on the formalization of thermodynamics under a dynamical systems perspective. The extension of thermodynamic principles—particularly the SLT—to arbitrary physical domains has attracted significant attention in recent years. Readers are referred to the work of van der Schaft [15] connecting the SLT to the system property of cyclo-dissipativity and evidencing SLT-like restrictions to power conversion in systems outside the thermodynamic domain [16].

Our previous works [17,18,19,20] introduced entropy-based formulations for power conversion optimization in multidomain systems, extending the applicability of SLT principles to interconnected systems that satisfy a version of the Clausius postulate restricted to periodic regimes. OCPs were formulated where *entropy generation* or *lost work* serve as a performance metric.

In this paper, we show that OCP based on entropy generation may be meaningfully formulated and solved for a general class of electromechanical systems. For these nonlinear multi-DoF systems, we recover essentially the same solution features as for the linear single-DoF electromechanical model of [17]: (i) the OCP solution requires velocity feedback, (ii) EGM yields a higher statistical expectation for the average harvested power in comparison with a controller that maximizes the FLT efficiency; and (iii) the unforced closed-loop system is semi-stable (velocity converges to zero while position converges to a point in the equilibrium manifold of the linkage, which depends on the initial conditions). Further, with bounded external forces, the closed-loop system exhibits bounded trajectories.

## 2. Background on the Cost Function

The main points behind the choice and construction of the cost function are summarized next. Readers are referred to  [17] for full details, and to  [18,19,20] for the application of these concepts to optimization and control of linear electromechanical systems and energy-aware robot trajectory planning.

**Figure 1:**
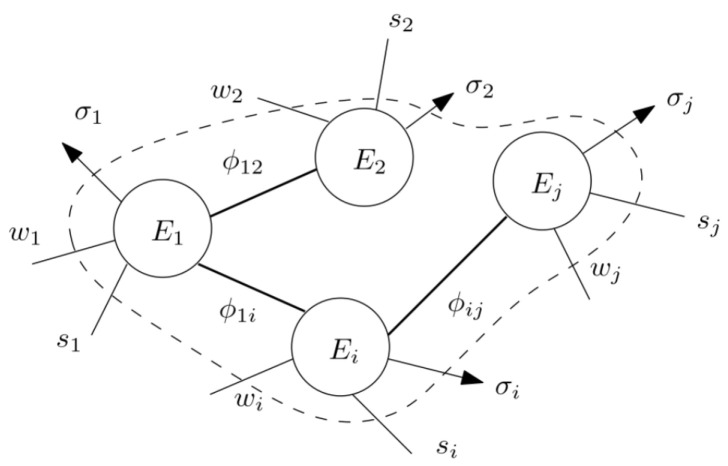
Interconnection of energy-storing dynamic systems [17].

Consider the interconnection of energy-storing dynamic systems as in Figure 1, where $E_{i}$ is the energy stored in subsystem *i*, $\phi_{ij}=-\phi_{ji}$ is the power received by *i* from *j*, $s_{i}$ is the external power exchanged by *i* (with sources capable of delivering or accepting power), $\sigma_{i}\geq 0$ is the power dissipated by *i* to the exterior, and $\omega_{i}$ is the rate of mechanical work exchanged between *i* and the exterior.

Each subsystem is assumed to satisfy a power balance equation among these quantities per the FLT at all times: ${\dot{E}}_{i}=s_{i}+\sum_{j=1}^{N}\phi_{ij}-\sigma_{i}-w_{i}=0$. In the classical thermodynamics setting, $\phi_{ij}$ represents heat flow and $E_{i}$ are functions of temperature. The Clausius postulate of the SLT states that heat cannot flow from a colder body towards a hotter body. In Haddad’s formalization of thermodynamics, this is used as an axiom to hold at all *t*:

(3) $$\left(E_{i}\left(t\right)-E_{j}\left(t\right)\right)\phi_{ij}\left(t\right)\leq 0,\;and\;\phi_{ij}=0⟺E_{1}\left(t\right)=E_{2}\left(t\right)$$

This postulate is the basis for the definition of *entropy generation* or *irreversible entropy production* in classical thermodynamics, a component of a system’s entropy change along irreversible processes such as frictional losses, heat transfer across finite temperature differences, and processes occurring outside equilibrium conditions [21].

However, the power transfer pattern of Equation (3) is not satisfied outside the thermal domain. Indeed the broad class of Hamiltonian systems, including those involving the mechanical, electrical and magnetic domains admit trajectories where power is directed “backwards”, i.e., from a low-energy subsystem towards a high-energy subsystem at particular instants of time. This presents an obstacle for the definition of entropy and the limitations it imposes in extended domains.

To circumvent this obstacle and the absence of a temperature variable from non-thermal engineering models, we consider cyclic trajectories and averaged forms of energy and power. Our work shows that a version of Equation (3) termed energy cyclo-directionality (ECD) [17,18,19] may hold in extended physical domains. For periodic trajectories, each subsystem in Figure 1 must also satisfy the *average* power balance:

(4) $${\bar{s}}_{i}+\sum_{j=1,j\neq i}^{N}{\bar{\phi }}_{ij}-{\bar{\sigma }}_{i}-{\bar{w}}_{i}=0$$

where ${\bar{s}}_{i}=\frac{1}{T}\int_{0}^{T}s\left(y\left(t\right),u\left(t\right),t\right)dt$ is the average cyclic integral of $s_{i}$ over a cycle and similar definitions apply for the other averaged variables. Note that ${\bar{\dot{E}}}_{i}=0$. The energy cyclo-directionality property is now defined.

**Definition** **1.** *Let P denote a set of periodic trajectories of the interconnected system. The interconnected system is said to satisfy energy cyclo-directionality (ECD) in P if there are constants $\beta_{i,j}\geq 0$ such that*

(5) $$\left({\bar{d}}_{i}-{\bar{d}}_{j}\right){\bar{\phi }}_{ij}\leq 0$$

*for all trajectories in P, where ${\bar{d}}_{i}=\beta_{i}{\bar{E}}_{i}$*

Note that ${\bar{d}}_{i}\left(t\right)$ play the role of generalized temperatures. Constants $\beta_{i}$ must be calculated for every specific system to guarantee this inequality. For subsystems with linear dynamics, this has been shown to reduce to the generalized eigenvalue problem [19], or, to $H_{\infty }$ norm calculations [17].

### Entropy Generation and Lost Work

In thermodynamics, the Clausius postulate leads to the definition of entropy generation, a non-negative quantity widely used to describe the deviation of a process from ideality (which involves reversibility and equilibrium). EGM techniques [22] involve the minimization of this quantity towards the efficient design and operation of thermodynamic systems. In our generalization [17], entropy generation takes the same form as its classical counterpart:

(6) $${\bar{X}}_{g}=\sum_{i=1}^{N}\sum_{j=1,j\neq i}^{N}\frac{{\bar{\phi }}_{ij}\left({\bar{d}}_{j}-{\bar{d}}_{i}\right)}{{\bar{d}}_{i}{\bar{d}}_{j}}$$

This cost function is non-negative whenever ECD holds. It coincides with the notion of *irreversible entropy production* [23], suitably extended to multidomain systems and restricted to periodic regimes.

Gouy and Stodola [7] linked entropy generation to the efficiency of heat engines through *lost work*, which quantifies the work lost due to irreversibility as follows:

(7) $$W_{lost}=T_{0}\Delta S_{gen}$$

where $T_{0}$ is the fixed environment temperature. This formula, known as the *Gouy–Stodola theorem*, is the foundation of SLT-based optimization of thermodynamic processes [6,22].

In our generalization, an arbitrary subsystem *r* is chosen to act as the reference, paralleling the role of the reference temperature in classical irreversible thermodynamics:

(8) $${\bar{W}}_{l,r}=d_{r}{\bar{X}}_{g}$$

Note that $d_{r}$ is constant for each periodic trajectory. The cost function used in the following sections is obtained from ${\bar{W}}_{l,r}$ by a convenient choice of reference subsystem.

## 3. Mathematical Model and Power Balances

**Figure 2:**
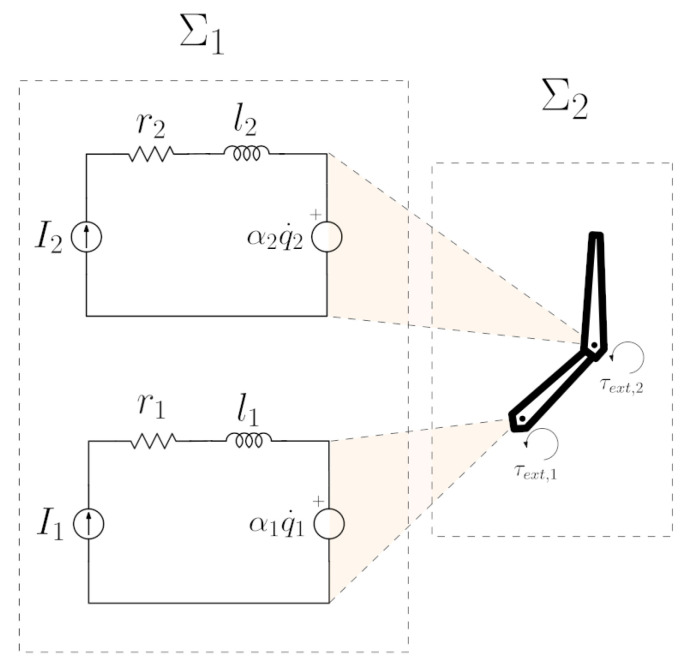
Multi-DoF linkage with current-driven actuators.

We consider an *n*-DoF serial linkage equipped with current-driven actuators as shown in Figure 2 for the case n=2.

With $u_{k}=I_{k}$, the dynamic equation of the $k_{\mathrm{th}}$ electric drive is

(9) $$V_{k}-u_{k}r_{k}-l_{k}{\dot{u}}_{k}-\alpha_{k}{\dot{q}}_{k}=0$$

where $V_{k}$, $r_{k}$, and $l_{k}$ are the voltage, electrical resistance, and inductance, respectively, and $\alpha_{k}$ is the back-EMF/torque constant. It is assumed that the electrical current can be directly controlled. The control of the power take-off (PTO) system in an energy harvesting device is a complex problem and has been extensively studied in the literature [24]. The present work focuses specifically on the mechanical-to-electrical energy conversion stage of the harvesting process. The governing equation of motion for the linkage under external torque $\tau_{ext}$ and control currents in the vector *u* is

(10) $$M\left(q\right)\ddot{q}+C\left(q,\dot{q}\right)\dot{q}+g\left(q\right)+f\left(\dot{q}\right)=Au+\tau_{ext}\left(t\right)$$

where *q* and $\dot{q}$ are the generalized joint coordinates and velocities, and M(q), $C(q,\dot{q})$, g(q) and $f(\dot{q})$ are respectively the mass matrix, the Coriolis/centripetal matrix, the gravity torque and the friction torque. Also, $A=\mathrm{diag}\left\{\alpha_{i}\right\}$, and $\tau_{ext}\left(t\right)$ is the external torque injecting mechanical power. Defining state variables $z_{1}=q$, $z_{2}=\dot{q}$ and $z={\left[z_{1}\;\;z_{2}\right]}^{T}$, the state-space model of the mechanical subsystem is

(11) $$\left\{\begin{array}{c} {\dot{z}}_{1}=z_{2}\;\\ {\dot{z}}_{2}=M{\left(z_{1}\right)}^{-1}\left\{Au-C\left(z\right)z_{2}-g\left(z_{1}\right)-f\left(z_{2}\right)+\tau_{ext}\left(t\right)\right\} \end{array}\right.$$

According to the framework of the previous section, the relevant average power and energy variables over a period *T* are as follows: the average mechanical dissipation is ${\bar{\sigma }}_{1}=\frac{1}{T}\oint_{0}^{T}u^{⊤}Ru\;dt$, where R=diag$\left\{r_{i}\right\}$, and the average electrical power of all sources is

(12) $$\begin{array}{c} {\bar{S}}_{1}=\frac{1}{T}\oint_{0}^{T}\sum_{k=1}^{n}\left(\alpha_{k}{\dot{q}}_{k}u_{k}+u_{k}^{2}r_{k}-l_{k}{\dot{u}}_{k}u_{k}\right)\;dt\\ =\frac{1}{T}\oint_{0}^{T}u^{⊤}Ru+{\dot{q}}^{⊤}Au\;dt \end{array}$$

The equation above is simplified using the fact that for periodic motions u(0)=u(T), so

(13) $$\begin{array}{c} \frac{1}{T}\oint_{0}^{T}\sum_{k=1}^{n}l_{k}{\dot{u}}_{k}u_{k}\;dt=\frac{1}{T}\sum_{k=1}^{n}\oint_{0}^{T}l_{k}{\dot{u}}_{k}u_{k}\;dt\\ =\frac{1}{T}\sum_{k=1}^{n}l_{k}\oint_{u(0)}^{u(T)}u_{k}du_{k}=0 \end{array}$$

The average stored magnetic energy is ${\bar{E}}_{1}=\frac{1}{T}\oint_{0}^{T}u^{⊤}Lu\;dt$, with L=diag$\left\{l_{i}\right\}$.

For the mechanical subsystem, the average dissipation is ${\bar{\sigma }}_{2}=\frac{1}{T}\oint_{0}^{T}{\dot{q}}^{⊤}f\left(\dot{q}\right)\;dt$, where *f* is assumed to satisfy ${\dot{q}}^{⊤}f\left(\dot{q}\right)\geq 0$ to represent power losses. The average rate of work is $\bar{W}=\frac{1}{T}\oint_{0}^{T}{\dot{q}}^{⊤}\tau_{ext}\;dt$ and the average stored mechanical energy is ${\bar{E}}_{2}=\frac{1}{T}\oint_{0}^{T}\left(\frac{1}{2}{\dot{q}}^{⊤}M\left(q\right)\dot{q}+P\left(q\right)\right)dt$, where P(q) is the potential energy of the linkage.

Multiplying Equation (10) by ${\dot{q}}^{⊤}$ and using the skew-symmetry property $z_{2}^{T}\left(\dot{M}-2C\right)z_{2}=0$ [25] yields the instantaneous power balance:

(14) $$\dot{K}+\dot{P}+{\dot{q}}^{⊤}f={\dot{q}}^{⊤}Au+{\dot{q}}^{⊤}\tau_{ext}$$

where *K* and *P* are the kinetic and potential energies, with $E_{2}=K+P$.

This shows that the instantaneous power exchanged across the electrical and mechanical subsystems is ${\dot{q}}^{⊤}Au$. However, to facilitate subsequent developments, we draw subsystem boundaries by regrouping terms in the power balance more conveniently. Add and subtract $S_{1}$ in Equation (14) and integrate over a cycle to obtain the average power balance of a subsystem denoted as $\Sigma_{2}$:

(15) $$0={\bar{S}}_{1}-{\bar{\phi }}_{12}-{\bar{\sigma }}_{2}-\bar{w}$$

where ${\bar{\phi }}_{12}={\bar{S}}_{1}-\frac{1}{T}\oint_{0}^{T}{\dot{q}}^{⊤}Au\;dt$ is defined and $\oint_{0}^{T}\dot{K}+\dot{P}\;dt=0$ was used. Rearranging Equation (12) yields the average power balance of a subsystem we denote as $\Sigma_{1}$:

(16) $$0={\bar{\phi }}_{12}-{\bar{\sigma }}_{1}$$

The rationale for this is that $\Sigma_{1}$ is *cyclo-passive* [15,26] with respect to supply rate $\phi_{12}$, that is ${\bar{\phi }}_{12}={\bar{\sigma }}_{1}\geq 0$ for all periodic trajectories. This greatly simplifies the enforcement of the ECD condition of Equation (5), which using $\beta_{1}=\gamma^{2}$, $\beta_{2}=1$ reduces to

(17) $$\gamma^{2}{\bar{E}}_{1}-{\bar{E}}_{2}\leq 0$$

for some constant $\gamma^{2}$.

### 3.1. ECD Property

To satisfy ECD, $\gamma^{2}$ should be chosen as $\gamma^{2}\leq \gamma_{crit}^{2}$ where

(18) $$\gamma_{crit}^{2}=\underset{T,q}{\inf}\;\frac{{\bar{E}}_{2}}{{\bar{E}}_{1}}=\underset{T,q}{\inf}\;\frac{\oint \frac{1}{2}{\dot{q}}^{⊤}M\left(q\right)\dot{q}+P\left(q\right)\;dt}{\oint u^{⊤}Lu\;dt}$$

where the infimum is calculated over all periods and cyclic trajectories. The value of this ratio depends on the physical characteristics of both the actuators and the mechanical components of the system. A numerical approach is generally required for the computation of $\gamma_{crit}^{2}$, although variational methods have also been considered [20]. However, the results of Section 4.2 show that practical, suboptimal velocity feedback gains may be calculated that are independent of $\gamma^{2}$, and only the existence of such a constant is assumed.

### 3.2. Lost Work and Cost Function

Using Equation (8) with r=2 and $d_{1}=\gamma^{2}{\bar{E}}_{1}$ and $d_{2}={\bar{E}}_{2}$, the lost work is

(19) $${\bar{W}}_{l,2}=d_{2}\left(\frac{d_{2}-d_{1}}{d_{1}d_{2}}\right){\bar{\phi }}_{12}=\left({\bar{E}}_{2}-\gamma^{2}{\bar{E}}_{1}\right)\frac{{\bar{\sigma }}_{1}}{{\bar{E}}_{1}},$$

Substituting the above average energy and dissipation terms gives

(20) $${\bar{W}}_{l,2}=\left({\bar{E}}_{2}-\gamma^{2}{\bar{E}}_{1}\right)G$$

where

(21) $$G=\frac{\oint_{0}^{T}u^{⊤}Ru\;dt}{\oint_{0}^{T}u^{⊤}Lu\;dt}.$$

is a Rayleigh quotient with bounds:

(22) $$\min_{i}\left\{\frac{r_{i}}{l_{i}}\right\}\leq G\leq \max_{i}\left\{\frac{r_{i}}{l_{i}}\right\}.$$

To relax the OCP, *G* is replaced by its upper bound, and therefore omitted. This leads to the cost function to be adopted:

(23) $$J=\frac{1}{T}\oint_{0}^{T}\left[\frac{1}{2}{\dot{q}}^{T}M\left(q\right)\dot{q}+P\left(q\right)-\frac{1}{2}\gamma^{2}u^{⊤}Lu\right]dt,$$

**Remark** **1.** *The choice of r=2 as reference subsystem is motivated solely by keeping the optimal control problem tractable. With this choice, several terms cancel out in Equation (19), including ${\bar{\phi }}_{12}$. The choices r=1 or attempting to minimize ${\bar{X}}_{g}$ instead of lost work would require strictly numerical solutions, reducing the interpretability of the results.*

## 4. OCP for Energy Harvesting and Its Solution

To impose constraints pertinent to energy harvesting, we introduce *n* auxiliary state variables $\xi_{i}$ corresponding to the electrical source power of each joint:

$${\dot{\xi }}_{i}=\frac{1}{T}\left(r_{i}u_{i}^{2}+\alpha_{i}z_{2,i}u_{i}\right)$$

and introduce the isoperimetric (integral) constraints $\xi_{i}\left(T\right)={\bar{s}}_{i}^{d}<0$, requiring each joint to produce net average harvested power in the cycle.

Using state variables, the OCP is formulated as

(24) $$\min J=\frac{1}{T}\oint_{0}^{T}\left[\frac{1}{2}z_{2}^{⊤}M\left(q\right)z_{2}+P\left(z_{1}\right)-\frac{1}{2}\gamma^{2}u^{⊤}Lu\right]dt$$

subject to

(25) $$\begin{array}{cc} \; & {\dot{z}}_{1}=z_{2},\\ \; & {\dot{z}}_{2}=M{\left(z_{1}\right)}^{-1}\left\{Au-C\left(z\right)z_{2}-g\left(z_{1}\right)-f\left(z_{2}\right)+\tau_{ext}\left(t\right)\right\},\\ \; & {\dot{\xi }}_{i}=\frac{1}{T}\left(r_{i}u_{i}^{2}+\alpha_{i}z_{2,i}u_{i}\right),\;\;i=1,\ldots ,n \end{array}$$

Boundary conditions:

(26) $$\left\{\begin{array}{c} z(0)=z(T),\\ \xi_{i}\left(0\right)=0,\;\xi_{i}\left(T\right)={{\bar{s}}_{i}}^{d}, \end{array}\right.$$

where ${{\bar{s}}_{i}}^{d}$ are given constants.

### 4.1. Solution

The standard variational approach to optimal control is employed [27]. The Hamiltonian is

(27) $$\begin{array}{cc} H & =\frac{1}{T}\left\{\frac{1}{2}z_{2}^{⊤}M\left(z_{1}\right)z_{2}+P\left(z_{1}\right)-\gamma^{2}u^{⊤}Lu\right\}+\lambda_{1}^{⊤}z_{2}\\ \; & \;+\lambda_{2}^{⊤}M^{-1}\left(z_{1}\right)\left\{Au-C\left(z\right)z_{2}-g\left(z_{1}\right)-f\left(z\right)+\tau_{ext}\left(t\right)\right\}\\ \; & \;+\frac{1}{T}\sum_{i=1}^{n}\lambda_{3,i}\left(r_{i}u_{i}^{2}+\alpha_{i}z_{2,i}u_{i}\right) \end{array}$$

where $\lambda_{1}$, $\lambda_{2}$, and $\lambda_{3,i}$ are the costate variables (Lagrange multipliers) associated with the dynamic constraints on $z_{1}$, $z_{2}$, and $\xi_{i}$, respectively.

Necessary conditions: Enforcing ∂H/∂u=0 gives

(28) $$-\frac{1}{T}\gamma^{2}Lu+A^{⊤}M^{-⊤}\left(z_{1}\right)\lambda_{2}+\frac{1}{T}\lambda_{3}\circ \left(2Ru+Az_{2}\right)=0$$

where ∘ denotes the Hadamard (element-wise) product of vectors. We require stationarity of the Hamiltonian with respect to $\tau_{ext}$:

(29) $$\frac{\partial H}{\partial \tau_{ext}}=-M^{-⊤}\left(z_{1}\right)\lambda_{2}=0.$$

Since $M(z_{1})$ is nonsingular, the above condition implies $\lambda_{2}=0$, and consequently ${\dot{\lambda }}_{2}=0.$ Note that $\frac{\partial^{2}H}{\partial \tau_{ext}^{2}}=0$; thus, *H* admits neither a minimum nor a maximum relative to $\tau_{ext}$. However, the stationarity condition enables the solution to proceed without knowledge of $\tau_{ext}$. This can be seen as a degenerate case of a min-max problem where the controller seeks to minimize the Hamiltonian for the worst-case external force. In our approach, the external torque is treated as neither cooperative nor adversarial.

Substituting $\lambda_{2}=0$ into Equation (28) shows that the optimal control must be a decoupled velocity feedback:

(30) $$u^{*}=-Fz_{2},\;\;\;\mathrm{where}\;\;\;F=diag\left\{\frac{\lambda_{3,i}\alpha_{i}}{2\lambda_{3,i}r_{i}-\gamma^{2}l_{i}}\right\}$$

For $u^{*}$ to minimize *H*, the second derivative of the Hamiltonian with respect to *u* must be positive definite:

(31) $$\frac{\partial^{2}H}{\partial u^{2}}>0⟹2\lambda_{3,i}r_{i}-\gamma^{2}l_{i}>0$$

The corresponding costate (adjoint) equations are derived as

(32) $$\begin{array}{ccc} {\dot{\lambda }}_{1} & = & -\frac{\partial H}{\partial z_{1}}=-\frac{1}{T}\frac{\partial }{\partial z_{1}}\left(\frac{1}{2}z_{2}^{⊤}M\left(z_{1}\right)z_{2}+P\left(z_{1}\right)\right)\\ {\dot{\lambda }}_{2} & = & -\frac{\partial H}{\partial z_{2}}=-\frac{1}{T}\frac{\partial }{\partial z_{2}}\left(\frac{1}{2}z_{2}^{⊤}M\left(z_{1}\right)z_{2}\right)-\lambda_{2}^{T}\frac{d{\dot{z}}_{2}}{dz_{2}} \end{array}$$

(33) $$\begin{array}{ccc} & - & \lambda_{1}-\frac{1}{T}\lambda_{3}\circ \left(Au\right) \end{array}$$

(34) $$\begin{array}{ccc} {\dot{\lambda }}_{3} & = & -\frac{\partial H}{\partial \xi }=0 \end{array}$$

Equation (34) shows that $\lambda_{3}$ is a constant, which from Equation (31) must satisfy the inequality

(35) $$\begin{array}{c} \lambda_{3,i}>\frac{\gamma^{2}l_{i}}{2r_{i}}\;i=1,2,\ldots ,n, \end{array}$$

For the remaining costates, substitute $\lambda_{2}={\dot{\lambda }}_{2}=0$ into Equation (33) and solve for $\lambda_{1}$, yielding

(36) $$\lambda_{1}=-\frac{1}{T}\frac{\partial }{\partial z_{2}}\left(\frac{1}{2}z_{2}^{⊤}M\left(z_{1}\right)z_{2}\right)-\frac{1}{T}\lambda_{3}\circ \left(Au\right)$$

Differentiating Equation (36) with respect to time and equating the result to the right-hand side of Equation (32) provides an explicit relationship between the time evolution of $\lambda_{1}$ and the system’s generalized coordinates:

(37) $$\begin{array}{cc} \; & -\frac{\partial }{\partial z_{1}}\left(\frac{1}{2}z_{2}^{⊤}M\left(z_{1}\right)z_{2}+P\left(z_{1}\right)\right)=\\ \; & -\frac{d}{dt}\frac{\partial }{\partial z_{2}}\left(\frac{1}{2}z_{2}^{⊤}M\left(z_{1}\right)z_{2}\right)+\lambda_{3}\circ \left(AF{\dot{z}}_{2}\right) \end{array}$$

From the Euler–Lagrange equation for a system with kinetic energy $\frac{1}{2}z_{2}^{⊤}M\left(z_{1}\right)z_{2}$ and potential energy $P(z_{1})$, we have

(38) $$\begin{array}{cc} \frac{d}{dt}\frac{\partial }{\partial z_{2}}\left(\frac{1}{2}z_{2}^{⊤}M\left(z_{1}\right)z_{2}\right)- & \frac{\partial }{\partial z_{1}}\left(\frac{1}{2}z_{2}^{⊤}M\left(z_{1}\right)z_{2}+P\left(z_{1}\right)\right)\\ \; & =M\left(z_{1}\right){\dot{z}}_{2}+C\left(z\right)z_{2}-g \end{array}$$

Combining Equations (37) and (38) gives

(39) $$\left(M(z_{1})-Q\right){\dot{z}}_{2}+C\left(z\right)z_{2}-g=0$$

where

$$Q=diag\left\{\frac{\lambda_{3,i}^{2}\alpha_{i}^{2}}{\;2\lambda_{3,i}r_{i}-\gamma^{2}l_{i}}\right\}$$

The necessary conditions of optimality require that trajectories $z^{*}\left(t\right)$ satisfy the condition defined by differential Equation (39) for some constants $\lambda_{3,i}$. Equation (39) corresponds to the dynamics of an *unforced* linkage with a modified mass matrix M−Q, where the principal moments of inertia are reduced by $Q_{ii}$ and gravity is reversed. Premultiplying this equation by $z_{2}^{⊤}$ and using the skew-symmetry once more, we obtain

(40) $$\dot{K}-\dot{P}=\frac{1}{2}\frac{d}{dt}\left(z_{2}^{⊤}Qz_{2}\right)$$

Integrating over time gives

(41) $$K-P=\frac{1}{2}z_{2}^{⊤}Qz_{2}+\mathrm{constant}$$

The above equation describes the set of integral curves where optimal trajectories must lie. Equation (40) corresponds to unforced motion which must be periodic. This optimality requirement is very restrictive. It may be satisfied for precise combinations of initial conditions, $\lambda_{3,i}$ and *T*. In addition, the solution requires such trajectories to produce harvesting, i.e., ${\bar{s}}_{i}^{d}<0$ in Equation (26).

### 4.2. Suboptimal Velocity Feedback and Harvested Power

Since decoupled velocity feedback is a necessary condition for optimal control, we designate trajectories resulting from applying the feedback of Equation (30) for arbitrary $\lambda_{3}$ satisfying (35) and $\gamma^{2}$ chosen to satisfy ECD as *suboptimal*. However, suboptimal trajectories need not satisfy Equation (39) under a given external torque.

In this section, we quantify harvesting, examine the asymptotic behavior of the cost function under suboptimal velocity feedback, and establish closed-loop stability properties, providing the main results of the paper formulated as three lemmas.

**Lemma** **1.** *Suppose the velocity feedback of Equation (30) is applied to the system in Equation (11), where $\gamma^{2}$ satisfies Equation (18) and $\lambda_{3}$ satisfies Equation (35). Then*

*i*. *A sufficient condition to produce harvesting (${\bar{s}}_{i}<0$) is given by*
(42) $$\lambda_{3,i}>\frac{\gamma^{2}l_{i}}{r_{i}}\;\;\;i=1,2,...,n$$
*ii*. *The control gains that maximize average harvested power are obtained at $\gamma^{2}=0$, yielding the control inputs*
$$u_{i}^{*}=-\frac{\alpha_{i}}{2r_{i}}z_{2i}$$

**Proof.**  The proof follows by straightforward substitutions and calculus. For [i.], substitute the velocity feedback law into Equation (12) and note that matrices *A*, *F*, and *R* are diagonal. The following expression for ${\bar{S}}_{1}$ is obtained:

(43) $$\begin{array}{c} {\bar{S}}_{1}=\frac{1}{T}\oint_{0}^{⊤}z_{2}^{⊤}\Lambda z_{2}\;dt \end{array}$$

where the constant matrix Λ is defined as

(44) Λ=FRF−AF

Then a negative-definite Λ guarantees harvesting both for the total system (${\bar{S}}_{1}<0$) as well as for each joint (as long as the periodic motion is non-trivial: $z_{2}\neq 0$). Substituting the expression for *F* into Equation (44) yields

(45) $$\Lambda =diag\left\{-\frac{\lambda_{3,i}\;\alpha_{i}^{2}\left(\lambda_{3,i}r_{i}-\gamma^{2}l_{i}\right)}{{\left(2\lambda_{3,i}r_{i}-\gamma^{2}l_{i}\right)}^{2}}\right\}$$

From Equation (45), it follows that Λ will be negative definite if the following inequality is satisfied:

$$\lambda_{3,i}>\frac{\gamma^{2}l_{i}}{r_{i}}\;\;\;i=1,2,...,n$$

This condition overrides the convexity constraint of Equation (35) and provides a sufficient criterion for the feasibility of energy harvesting.

To establish [ii.], calculus is used to analyze the dependence of Λ on $\gamma^{2}$. To maximize harvesting, the components of this matrix must be minimized. Taking the derivative of $\Lambda_{ii}$ with respect to $\gamma^{2}$ and setting it to zero, we obtain $\gamma^{2}=0$, for which

(46) $$\Lambda^{min}=diag\left\{-\frac{\alpha_{i}^{2}}{4r_{i}}\right\}$$

It can be checked that this is indeed a minimum. Substituting this value into Equation (45) gives $u_{i}^{*}$ in the lemma.    □

**Remark** **2.** *The above is based on the observation that $\gamma^{2}$ may be freely chosen, as long as it satisfies the ECD condition (18). Also, the isoperimetric constraint on the harvested energy requires that this value equals the desired targets ${{\bar{s}}_{i}}^{d}<0$. In principle, a value for $\lambda_{3}$ could be calculated by solving Equation (43) for an arbitrary desired value of the harvested energy ${{\bar{S}}_{1}}^{d}$. Since $z_{2}\left(t\right)$ depends on the choice of $\lambda_{3}$, the initial conditions and the external torques, this would require a numerical procedure involving simulation and an assumed external torque history. In contrast, Lemma 1 provides information on the achievable value of ${\bar{s}}_{i}$ without information on the external torque $\tau_{ext}$ or its spectral characteristics. For $\gamma^{2}=0$, ECD is trivially satisfied, and the cost (lost work) becomes proportional to the average mechanical energy of the linkage.*

Equation (30) shows that for $\gamma^{2}=0$, the control input no longer depends on $\lambda_{3}$. It depends only on the back-EMF constant and the electrical resistance. This result aligns closely with the findings of [28], where a similar dependence appears in the context of risk-sensitive control of a linear energy harvesting system when a strong emphasis is put on the steadiness of the harvested power within the cost function. It can also be observed that the controller adds damping to the system. These results are also consistent with our findings in [29], where an optimal velocity feedback was obtained to maximize power regenerated by a powered prosthetic leg (a 2-link planar mechanism) during the swing phase.

### 4.3. Horizontal Linkages

For a horizontal linkage P=0, and we obtain additional insight into the asymptotic behavior of the OCP solutions as summarized in the following lemma.

**Lemma** **2.** *Suppose g(q)=0 in Equations (10) and (30) is applied, where $\gamma^{2}$ satisfies Equation (18) and $\lambda_{3}$ satisfies Equation (35). Suppose also that z(t) satisfies (39). Then:*

*i*. *The OCP cost function adopts a global minimum when*
(47) $$\lambda_{3,i}=\frac{\gamma^{2}l_{i}}{r_{i}}.$$
  *which corresponds to the case ${\bar{S}}_{1}=0$.*
*ii*. *A control satisfying all necessary conditions for optimality and ${\bar{S}}_{1}>0$ does not exist. However,*
$$\lim_{\lambda_{3,i}\to \infty }{\bar{S}}_{1}=\frac{1}{T}\oint_{0}^{⊤}z_{2}^{⊤}\Lambda^{min}z_{2}\;dt$$
  *where $\Lambda^{min}$ achieves maximum harvesting per Equation (46). That is, maximum harvesting is achieved either with $\gamma^{2}=0$ or with $\lambda_{3,i}\to \infty$.*
*iii*. *For $\gamma^{2}=0$, the cost satisfies*
$$\lim_{\lambda_{3,i}\to 0}J=0$$

**Proof.**  If $P(z_{1})=0$, Equation (41) simplifies to

(48) $$K=\frac{1}{2}z_{2}^{⊤}Qz_{2}+\mathrm{constant}.$$

Substituting *K* above and $u^{*}$ from Equation (30), the optimal cost becomes

(49) $$J^{*}=\frac{1}{2T}\oint_{0}^{T}z_{2}^{⊤}\left(Q-\gamma^{2}FLF\right)z_{2}\;dt$$

The minimum cost occurs when $Q-\gamma^{2}FLF=0$, which, when solved, gives the closed-form solution for $\lambda_{3,i}$ in [i].

To show [ii.], note that two conditions restrict $\lambda_{3,i}$: the convexity condition in Equation (35) and the harvesting condition of Equation (42):

(50) $$\left\{\begin{array}{c} \lambda_{3,i}>\frac{\gamma^{2}l_{i}}{2r_{i}},\;(\mathrm{Hamiltonian}\;\mathrm{convexity})\\ \lambda_{3,i}>\frac{\gamma^{2}l_{i}}{r_{i}},\;(\mathrm{Harvesting}\;\mathrm{feasibility}). \end{array}\right.$$

Clearly the condition corresponding to minimum cost of [i.] is incompatible with the requirement for energy harvesting. The only possible case that allows energy harvesting is $\gamma^{2}=0$. Under this condition, the convexity requirement simplifies to

(51) $$2\lambda_{3,i}r_{i}>0,$$

which indicates that the convexity of the Hamiltonian diminishes as $\lambda_{3,i}$ approaches zero. Consequently, an optimal control does not exist (in the sense that the limiting value of the cost is an infimum, not a minimum). The limit in [ii.] is directly evaluated from Equation (45), and [iii.] also follows from previous considerations.    □

**Remark** **3.** *Lemma 2 indicates that either $\gamma^{2}=0$ or $\lambda_{3,i}\to \infty$ enhance energy harvesting, yielding the same limiting control gains and maintaining the convexity of the Hamiltonian. As $\lambda_{3,i}\to 0$, condition (42) implies $\gamma^{2}\to 0$ also. Thus, per [iii.] the objective function is minimized by reducing $\lambda_{3,i}$ towards zero. In the limit, the pair $\left(\gamma^{2},\lambda_{3,i}\right)=\left(0,0\right)$ leads to Q=0, and therefore F=0. Since the kinetic energy is positive definite, this requires $z_{2}\to 0$.*

This implies that achieving minimal lost work (or equivalently minimal entropy generation) requires the system to move “infinitely slowly”, or “quasi-statically”, to use the terminologies of classical thermodynamics. Such “reversible” processes incur no entropy generation. The notion of a quasi-static trajectory has been indeed put in a solid footing by Haddad [14] using the language of dynamical systems. Essentially, such trajectories are those contained in the equilibrium manifold.

### 4.4. Closed-Loop Stability

Under periodic and bounded external torques, an energy harvester must produce bounded trajectories. Consider first the unforced closed-loop system. Since velocity feedback injects damping, stability follows immediately by considering a Lyapunov function candidate given by the total mechanical energy of the linkage:

$$V\left(z\right)=\frac{1}{2}z_{2}^{⊤}M\left(z_{1}\right)z_{2}+P\left(z_{1}\right),$$

whose derivative along the closed-loop dynamics is

(52) $$\dot{V}=-z_{2}^{⊤}f\left(z_{2}\right)-z_{2}^{⊤}Fz_{2}.$$

A standard argument based on LaSalle’s invariance principle shows that the unforced closed-loop trajectories converge to a point $\left({\bar{z}}_{1},0\right)$ on the equilibrium manifold that depends on the initial conditions. That is, the velocity feedback control produces closed-loop *semi-stability* [30]. Next, we consider $\tau_{ext}\neq 0$ and show that the closed-loop system is input-to-state stable (ISS). This property implies that the states remain bounded for bounded $\tau_{ext}$. This is readily established by first noting that the closed-loop system is *passive* with storage function V(z). Indeed, with external torque Equation (52) becomes

$$\dot{V}=-z_{2}^{⊤}f\left(z_{2}\right)-z_{2}^{⊤}Fz_{2}+z_{2}^{T}\tau_{ext}\leq z_{2}^{⊤}\tau_{ext}$$

and therefore for any $\left[t_{1},t_{2}\right]$

$$V\left(t_{2}\right)-V\left(t_{1}\right)\leq \int_{t_{1}}^{t_{2}}z_{2}^{T}\left(t\right)\tau_{ext}\left(t\right)\;dt$$

Intuitively, the left-hand side is the change in mechanical energy and the right-hand side is the energy injected by the external excitation. The inequality says that the mechanical system may not gain more energy than that provided by the input, implying boundedness of states for bounded inputs. A formal statement of the link between passivity and ISS may be found, for instance, in [31]. Note that velocities remain bounded due to passivity, but joint positions remain bounded modulo 2π, due to the configuration space of the robot being itself bounded.

## 5. Direct Efficiency Maximization

For comparison purposes, we summarize the solution of an OCP based on common efficiency, defined for harvesting (${\bar{S}}_{1}<0$) as $\eta_{1}=-{\bar{S}}_{1}/\left(-{\bar{S}}_{1}+\bar{\sigma }\right)$.  With isoperimetric constraints on the average harvested power as above, efficiency maximization is equivalent to total loss minimization, corresponding to the cost function:

(53) $$J_{\eta_{1}}=\bar{\sigma }=\frac{1}{2T}\int_{0}^{T}z_{2}^{T}f\left(z_{2}\right)+u^{T}Ru\;dt$$

An OCP is formulated with the same constraints and boundary conditions as above, assuming linear viscous friction. Following standard variational procedures, a closed-form solution is available as velocity feedback whose gains are calculated by solving quadratic equations. Readers are referred to [32] for the procedural details. The optimal control is shown to always produce closed-loop semi-stability and yield net harvesting. The optimal gains depend on the electrical parameters but are independent of the linkage parameters. Unlike our EMG solution, knowledge of friction coefficients is required.

## 6. Example and Simulation Studies

We first consider a single-pendulum energy harvester to illustrate the difficulties in achieving the conditions for the exact optimal solutions.

### 6.1. Case 1: Single-Pendulum Energy Harvester

For a single pendulum, the model of Equation (11) reduces to

(54) $$I_{c}\ddot{q}+b\dot{q}+mgl_{c}\sin q=\tau_{ext}+\alpha u,\;\;\;$$

where $I_{c}$ is the moment of inertia about the center of rotation, *b* is the viscous friction coefficient at the joint, and $l_{c}$ denotes the location of the center of mass of the link. The costate Equation (39) associated with the optimal control problem becomes

(55) $$\left(I_{c}-Q\right)\ddot{q}-mgl_{c}\sin q=0,\;\;\;$$

where *Q* is

$$Q=\frac{\lambda_{3}^{2}\alpha^{2}}{\;\left(2\lambda_{3}r-\gamma^{2}l\right)}$$

substituting $I_{c}\ddot{q}$ from the equation of motion into the costate equation yields the following equation for the optimal trajectories:

(56) $$Q\ddot{q}+b\dot{q}+2mgl_{c}\sin q=\tau_{ext}$$

This result shows that the optimal trajectories must satisfy the governing equation of a single pendulum with a modified inertia term and an effectively doubled gravitational term. The total harvested energy can be calculated as

(57) $$\bar{S}=\frac{\Lambda }{T}\oint_{0}^{T}{\dot{q}}^{2}\;dt$$

where

(58) $$\Lambda =diag\left\{-\frac{\lambda_{3}\;\alpha^{2}\left(\lambda_{3}r-T\gamma^{2}l\right)}{{\left(2\lambda_{3}r-T\gamma^{2}l\right)}^{2}}\right\}$$

The value of the constant costate $\lambda_{3}$ must be determined so that the isoperimetric constraint $\bar{S}={\bar{S}}^{d}$ in Equation (26) is met. However, the velocity trajectory $\dot{q}$ must be known to evaluate the harvested energy. This, in turn, requires prior knowledge of the external torque in the interval [0,T], which is inconsistent with the information available in energy harvesting applications. The suboptimal solution, in contrast, does not require this information and leads to an explicit gain that can be implemented in real time.

### 6.2. Simulation Study: Double-Pendulum Energy Harvester

**Figure 3:**
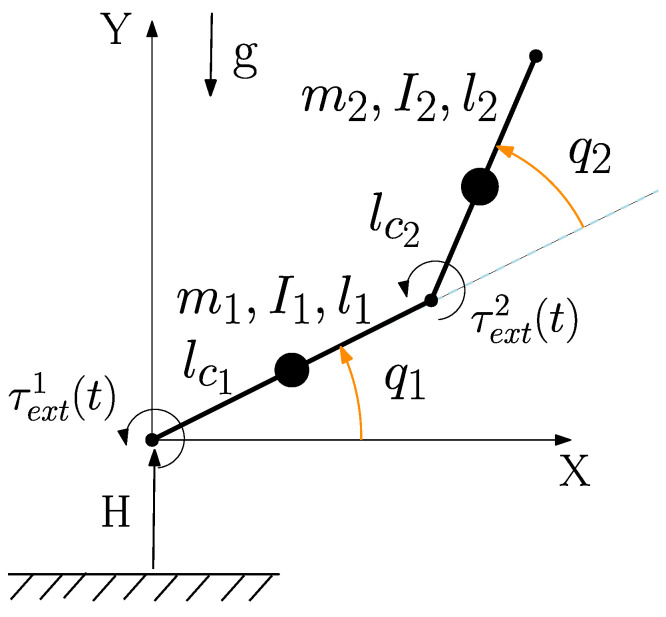
Schematic of the actuated double pendulum with external random periodic torques applied at the joints.

We demonstrate the benefits of the proposed approach using a two-link planar linkage as in Figure 3, for which the mass, Coriolis and gravity terms are widely available. Friction is assumed to be linearly viscous: $f\left(z\right)=Bz_{2}$, where B=diag$\left\{b_{i}\right\}$ contains non-negative coefficients. The numerical values of the mechanical and electrical parameters employed in the simulations are shown in Table 1 and also available in the code accompanying this paper.

The study has two aims: (i) to determine how the proposed suboptimal controller approximates the true optimal solution over a large number of trajectories that satisfy Equation (39), and (ii) to compare the harvesting performance of the proposed approach against the maximum efficiency controller under stochastic external torque inputs.

### 6.3. Optimal vs. Suboptimal Control Gains

A search for initial conditions z(0), periods T∈[010] and constants $\Lambda_{3}$ that resulted in periodic trajectories of Equation (39) (to a tolerance of 0.01 for ∥z(0)−z(T)∥) and with ${\bar{S}}_{1}\leq 1$ W was conducted. Since the periodic trajectories of Equation (39) may be unstable and sensitive to initial conditions, the process is computationally intensive. In total, 1450 such trajectories were computed using $\gamma^{2}=0.5$, and the corresponding exact gains *K* recorded. For the parameters of the study, K(1,1) and K(2,2) were narrowly distributed, with means of 5.06 and 5.02, respectively, and standard deviations of 0.185 and 0.004. The suboptimal gains calculated from Lemma 1 are both 5. This means that suboptimal gains are a good approximation to those generating the true optimal solutions.

### 6.4. Monte Carlo Study

To compare the harvesting performance and the efficiencies obtained with the suboptimal EGM gains or the maximum efficiency gains, a set of 1000 random periodic external torques was generated and tested with either controller, subject to the condition ${\bar{S}}_{1}\leq -1$ W. All $\tau_{ext}$ had T=5 s. To facilitate the computations, $q_{1}\left(t\right)$ and $q_{2}\left(t\right)$ were parameterized with 5-term Fourier series:

(59) $$\begin{array}{ccc} q_{1}\left(t\right) & = & X_{1}+X_{2}\cos\left(wt\right)+X_{3}\sin\left(wt\right)+X_{4}\cos\left(2wt\right)+X_{5}\sin\left(2wt\right) \end{array}$$

(60) $$\begin{array}{ccc} q_{2}\left(t\right) & = & X_{6}+X_{7}\cos\left(wt\right)+X_{8}\sin\left(wt\right)+X_{9}\cos\left(2wt\right)+X_{10}\sin\left(2wt\right) \end{array}$$

This guarantees periodicity, both for z(t) and $\tau_{ext}\left(t\right)$. For a random guess of Fourier coefficients $X_{i}$, z(t) and $\dot{z}\left(t\right)$ are evaluated from analytical expressions. The external torque is uniquely computed for a given trajectory using Equation (10) once a controller has been selected. All components of the average power balance may be computed and recorded for each episode of $\tau_{ext}\left(t\right)$ meeting the constraints, from which the performance statistics are generated. The generation of random trajectories was performed separately for each controller.

**Figure 4:**
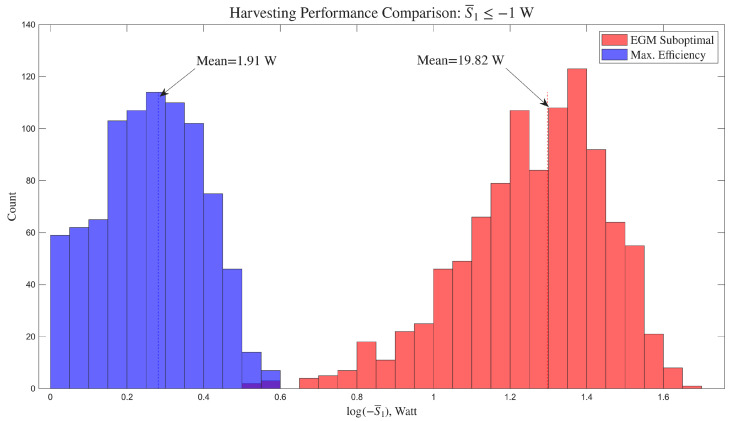
Distribution of average harvested power with randomly periodic external torques.

Figure 4 presents histograms (based on raw counts for each bin) that illustrate the statistical properties of each controller. The horizontal axis shows $\log(-{\bar{S}}_{1})$ for clarity of presentation. This is because the data for maximal efficiency is narrowly distributed around the mean, resulting in a much higher peak, while the data for EGM is more broadly distributed.

Histograms were generated using the histogram function in Matlab 2025a, which uses an automatic algorithm that returns bins of uniform width, chosen to cover the range of the data and reveal the underlying shape of the distribution. The histogram is normalized to approximate the underlying probability density function.

The EGM-based controller has markedly superior harvesting performance across the external torque input episodes. The expectation of the average harvested power is higher than that of the direct efficiency maximization method as clearly shown in the figure. The efficiency achieved with the EGM approach is, as expected, lower in comparison to that achieved using a controller that maximizes efficiency directly. Indeed, with EGM, the average efficiency is 49.9%, while the maximum efficiency controller yields an average of 95.1% for the assumed physical parameters.

The results are qualitatively the same when conducted with different system parameters or *T*; however, the amount of overlap between the distributions and their spread may change.

### 6.5. Non-Periodic Trajectories

While the conditions of true optimality depend on the period, this is not the case for the suboptimal gains as it can be observed in the feedback control gain formula. This means that no information about the periodicity of the excitation (further, no spectral information is required). This suggests that the same observations regarding the statistics of harvested power may apply to the non-periodic case. Using an argument similar to the treatment of non-periodic signals in Fourier analysis, we can regard the non-periodic signal as periodic by truncation and periodic extension, and then let the period go to infinity [33].

To demonstrate this argument, we have a collection of 1000 non-periodic joint trajectories of the form

(61) $$\begin{array}{ccc} q_{1}\left(t\right) & = & X_{1}+X_{2}\cos\left(wt\right)+X_{3}\sin\left(wt\right)+X_{4}\cos\left(3.1wt\right)+X_{5}\sin\left(1.5wt\right) \end{array}$$

(62) $$\begin{array}{ccc} q_{2}\left(t\right) & = & X_{6}+X_{7}\cos\left(wt\right)+X_{8}\sin\left(wt\right)+X_{9}\cos\left(2.6wt\right)+X_{10}\sin\left(1.7wt\right) \end{array}$$

where the coefficients were randomly selected subject to the condition ${\bar{S}}_{1}\leq -1$ W. As in the periodic case, the model was used to uniquely compute the external torques that produce these trajectories and the same information was collected and analyzed.

It should be noted that the net change in mechanical energy need not be zero for non-periodic trajectories since the mechanism is not required to return to the same configuration and joint velocities. This means that efficiency maximization is no longer equivalent to loss minimization. The comparison below is therefore between the suboptimal EGM controller and a loss minimization controller, which is the same as that used in the periodic case.

**Figure 5:**
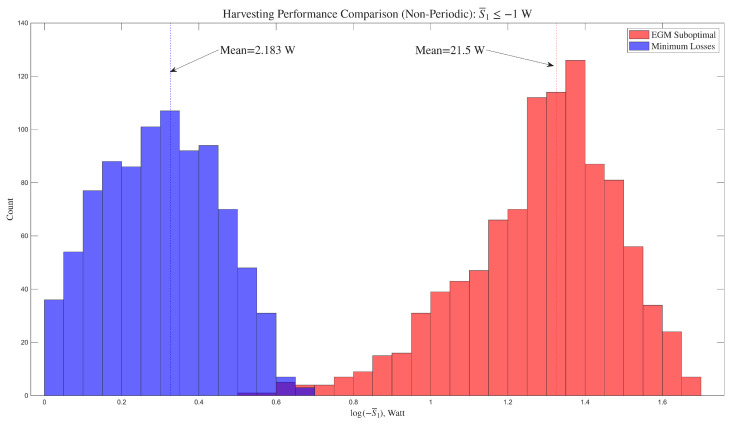
Distribution of average harvested power with randomly-generated non-periodic external torques.

As shown in Figure 5 and summarized in Table 2, the results show that for periodic and non-periodic trajectories, the suboptimal EGM solution produces a higher expectation for the average harvested power in comparison with loss minimization, while the efficiency associated with the latter controller is higher as expected due to direct loss minimization.

**Remark** **4.** *For periodic trajectories the net change of mechanical energy is zero and the efficiency is calculated as the ratio of harvested power to supplied work. For non-periodic trajectories, the mechanism will generally have a net change of stored mechanical energy in [0,T]. The efficiency may be calculated as before, or the denominator may be adjusted with the net change of mechanical energy, which is not regarded as a loss. The efficiencies reported above for the non-periodic case are an average of both forms.*

## 7. Conclusions and Future Work

The proposed cost function is based on, and recovers, the features of thermodynamic optimization, where true unconstrained optimality corresponds to an idealized process occurring along the equilibrium manifold. Indeed, for the case of horizontal linkages, achieving zero lost work requires system motion to become arbitrarily slow, mirroring the classical thermodynamic notion that reversible processes generate no entropy only when executed infinitely slowly. From a practical standpoint, OCPs defined with this cost function are analytically tractable and lead to suboptimal solutions that do not require information about the mechanical subsystem parameters, notably friction characteristics. No information about the external torque input or its spectrum is required.

The Monte Carlo study confirms that a controller derived from the proposed EGM approach will produce higher average harvested power over repeated episodes with random excitation, a feature which is highly relevant to energy harvesters. While the proposed controller is less efficient than one designed to maximize efficiency, harvesting applications reasonably prioritize larger amounts of harvested power over efficiency since the resource is abundant and essentially free. These outcomes are reminiscent of the Maximum Power Theorem [34], originally formulated for electrical networks. Indeed, the EGM solutions for the periodic and non-periodic cases produce an efficiency of 1/2, which had also been observed for the linear systems considered in our earlier work [17]. The results of this paper thus suggest a deep connection between maximum power transfer and entropy minimization, even in a generalized sense.

An immediate extension of our work is to “color” the excitation by modeling it as the output of a filter reflecting known spectral characteristics. This introduces additional dynamics, requiring a new solution. It is expected that the resulting controllers will offer superior performance over those derived with an unknown spectrum. Also, direct external torque inputs were assumed. For a practical harvesting application, external force must also be considered, which contributes a term $J^{T}\left(q\right)F_{ext}$, where J(q) is the kinematic Jacobian at the point of application of $F_{ext}$. This requires straightforward modifications.

Finally, the selection of subsystems used in deriving the lost work expression is somewhat arbitrary and was chosen primarily to simplify the analysis. Developing a systematic and general procedure for subsystem decomposition would be a valuable direction for future research.

## Figures and Tables

**Table 1:** Parameters used in the double-pendulum harvester model.

Parameter	Symbol	Value	Units
Link 1 mass	$m_{1}$	1	kg
Link 2 mass	$m_{2}$	0.5	kg
Link 1 CM	$l_{c1}$	0.5	m
Link 2 CM	$l_{c2}$	0.25	m
Link 1 length	$l_{1}$	0.5	m
Link 2 length	$l_{2}$	0.5	m
Link 1 inertia	$I_{1}$	0.15	kg-m^2^
Link 2 inertia	$I_{2}$	5	kg-m^2^
Link 1 torque constant	$\alpha_{1}$	1	N-m/A
Link 2 torque constant	$\alpha_{2}$	2	N-m/A
Link 1 resistance	$r_{1}$	0.1	Ω
Link 2 resistance	$r_{2}$	0.2	Ω
Link 1 inductance	$L_{1}$	0.01	H
Link 2 inductance	$L_{2}$	0.02	H
Joint 1 viscous friction	$b_{1}$	0.01	N-m-s
Joint 2 viscous friction	$b_{2}$	0.008	N-m-s

**Table 2:** Summary of Monte Carlo study statistics between EGM and loss minimization (LM).

Case	Mean Harvested Power	Std. Dev.	Mean Efficiency
EGM Periodic	19.98	7.66	0.499
EGM Non-periodic	21.93	9.12	0.499
LM Periodic	1.91	0.56	0.951
LM Non-periodic	2.21	0.75	0.996

## Data Availability

The Montecarlo studies can be reproduced by with the code posted in the GitHub repository https://github.com/csurowing/Generalized-Entropy-Generation-Minimization.
